# The origin, source, and cycling of methane in deep crystalline rock biosphere

**DOI:** 10.3389/fmicb.2015.00725

**Published:** 2015-07-17

**Authors:** Riikka Kietäväinen, Lotta Purkamo

**Affiliations:** ^1^Geological Survey of FinlandEspoo, Finland; ^2^VTT Technical Research Centre of FinlandEspoo, Finland

**Keywords:** abiotic methane, deep subsurface, Precambrian bedrock, carbon cycle, methanogenesis, methanotrophy, isotopic fractionation

## Abstract

The emerging interest in using stable bedrock formations for industrial purposes, e.g., nuclear waste disposal, has increased the need for understanding microbiological and geochemical processes in deep crystalline rock environments, including the carbon cycle. Considering the origin and evolution of life on Earth, these environments may also serve as windows to the past. Various geological, chemical, and biological processes can influence the deep carbon cycle. Conditions of CH_4_ formation, available substrates and time scales can be drastically different from surface environments. This paper reviews the origin, source, and cycling of methane in deep terrestrial crystalline bedrock with an emphasis on microbiology. In addition to potential formation pathways of CH_4_, microbial consumption of CH_4_ is also discussed. Recent studies on the origin of CH_4_ in continental bedrock environments have shown that the traditional separation of biotic and abiotic CH_4_ by the isotopic composition can be misleading in substrate-limited environments, such as the deep crystalline bedrock. Despite of similarities between Precambrian continental sites in Fennoscandia, South Africa and North America, where deep methane cycling has been studied, common physicochemical properties which could explain the variation in the amount of CH_4_ and presence or absence of CH_4_ cycling microbes were not found. However, based on their preferred carbon metabolism, methanogenic microbes appeared to have similar spatial distribution among the different sites.

## Introduction and historical perspective

Methane is a key compound in the global carbon cycle. In the shallow subsurface CH_4_ is mainy produced by anaerobic digestion of organic matter. Deeper in the geological strata CH_4_ is found in large quantities within sedimentary formations and unconventional resources, such as shale gas, have also proven to be important reserves of CH_4_ (Arthur and Cole, 2014). Other sources of CH_4_ include methane hydrates and clathrates in deep lake sediments and seafloor (Walter et al., 2006; Ruppel, 2011; Kretschmer et al., 2015; Treat et al., 2015). In addition, CH_4_ is a dominant gas in many Precambrian continental bedrock formations (Fritz et al., 1987; Karus et al., 1987; Nurmi et al., 1988; Sherwood Lollar et al., 1993a,b; Ward et al., 2004; Sherwood Lollar et al., 2006; Pitkänen and Partamies, 2007; Hallbeck and Pedersen, 2008a; Stotler et al., 2010; Kietäväinen et al., 2013, 2014). Table 1 describes the carbon geochemistry of several continental study sites in Fennoscandia, South Africa, and North America within the depth range from few hundred meters down to 3.4 km. Microbes involved in methane cycling have been detected in many of these sites.

**Table 1:** **Characteristics of deep Precambrian continental bedrock sites where microbes contributing to the methane cycle have been studied**.

**Site**	**Main rock types**	**Age (rock)**	**Depth^1^**	**T^1^**	**pH**	**Salinity^1^**	**CH^1^_4_**	**CH^1^_4_**	**δ^13^C_*CH4*_**	**δ^2^H^2^_*CH4*_**	**δ^13^C_*DIC*_**	**DIC**	**TOC**	**DOC**	**Methane-cycling microbes^6^**	**References^7^**
		**Ma**	**mbs**	**°C**		**TDS g L^−1^**	**mM**	**Vol-%**	**‰ VPDB**	**‰ VSMOW**	**‰ VPDB**	**mM**	**mM**	**mM**		
**CANADA**																
Lupin	Metagraywacke, slate, banded iron formation	2600	1130	11	7.9 …9.2	40	35	86.7	−56.1 …−42.4	−340/−324	−5.2 …10.5	0.088 …0.21	0.12 …0.33	0.15 …0.36	ND	26, 37, 38
**FINLAND**																
Hästholmen	Rapakivi granite	1630	985		6.8 …8.5	31.8	0.009	0.09	−63 …−28		−21.5 …−11.5	0.38 …2.22	0.30 …0.34	0.08 …0.82	ND	10, 20
Kivetty	Granite, granodiorite	1880	855		7.8 …9.0	0.23	0.006	0.09	−75.5 …−15		−24.2 …−18.3	0.99 …2.15	0.18	0.14 …1.27	ND	2, 10, 32
Olkiluoto	Migmatitic gneiss, mica gneiss, granite	1850	960	20	7.8 …8.3	84	41.4	80.5	−63.5 …−22.4	**^3^		0.04 …5.9	0.12 …0.14		MG, MT, ANME	3, 10, 24, 30
Outokumpu	Mica schist, black schist, granodiorite, serpentinite	1900	2480	40	8.4 …10.1	68.9	32.3	80	−31.2 …−24.4	−283/−279					MG, MT	11, 15, 25, 33, 34, 35
Palmottu	Garnet-cordierite gneiss, granite	1900	417		6.2 …9.4	1.6					−28.6 …−6.1	0.5 …3^4^			ND	1, 12, 29
Romuvaara	Tonalite gneiss	2700	566		8.4	0.17	0.003	0.2	−59.5 …−37			1.85	1.05		ND	10, 31
**SOUTH AFRICA**																
Beatrix	Conglomerate, quartzite	2900	1390	40	7.7 …7.9		35.2	89.6	−52.8 …−43.5	−195/−214	−32	0.34	0.14 …0.18	0.14	MG	4, 6, 18, 39
Driefontein	Andesite, quartzite	2700	3300	43	6.0 …7.4		17.5		−55.5 …−40.2	−218/−368	−17.8 …−8.0	0.41 …0.57	0.40 …1.83	0.67	MG, ANME	4, 6, 18, 22, 23, 36
Evander	Conglomerate, quartzite	2900	2230	45	7.2 …8.6		15.8	78.8	−61.2 …−40.7	−211/−281			0.22 …0.61		MG, ANME	6, 18, 39
Kloof	Andesite	2700	3400	59	7.6 …8.5		29.8	57.3	−40 …−28.7	−253/−300	−14.7	0.02^5^	0.46 …1.67		ND	14, 18, 36, 39
Mponeng	Metabasalt	2700	3300	52	9.3		20		−33.2 …−27.8	−390/−349			0.43		MG	6, 18, 19, 36
**SWEDEN**																
Äspö	Granite, granodiorite	1800	860	20	6.8 …7.8	16	1					0.16 …0.34^4^	0.11 …0.57	0.025 …1.5	MG, MT	5, 7, 13, 16, 17, 28
Forsmark	Meta-granite	1900	1002		7.3 …8.3	15	0.21					0.04 …1.5^4^		0.12 …0.13	MG, MT	5, 7, 9
Laxemar	Granite, quartz monzodiorite	1800	922	20	7.5 …8.4	18	0.039	1.4				0.13 …5.4^4^	0.12 …1.67	0.12 …1.75	MG	7, 8, 9, 28
**USA**																
Homestake, SURF	Metasediments	2000	1478	33	6.6 …8.5		0.00037								MG, MT	21, 27

1 *Maximum values are given for depth, temperature (T), salinity and CH_4_ concentration*.

2 *For the same samples as min. and max. values of δ^13^C_CH4_*.

3 *Not determined for these particular samples. Overall range of δ^2^H_CH4_ in Olkiluoto is from −309 to −175 ‰ VSMOW*.

4 *HCO^−^_3_ concentration*.

5 *TIC*.

6 *ND, not detected; MG, methanogens; MT, aerobic methanotrophs; ANME, anaerobic methanotrophs*.

7 *[1] Ahonen et al., 2004, [2] Anttila et al., 1999, [3] Bomberg et al., 2015, [4] Borgonie et al., 2011, [5] Chi Fru, 2008, [6] Gihring et al., 2006, [7] Hallbeck and Pedersen, 2008a, [8] Hallbeck and Pedersen, 2008b, [9] Hallbeck and Pedersen, 2012, [10] Haveman et al., 1999, [11] Itävaara et al., 2011a, [12] Kaija et al., 1998, [13] Kalyuzhnaya et al., 1999, [14] Kieft et al., 2005, [15] Kietäväinen et al., 2013, [16] Kotelnikova and Pedersen, 1997, [17] Kotelnikova and Pedersen, 1998, [18] Lin et al., 2005a, [19] Lin et al., 2006b, [20] Luukkonen et al., 1999, [21] Morelli et al., 2010 and references therein, [22] Moser et al., 2003, [23] Moser et al., 2005, [24] Nyyssönen et al., 2012, [25] Nyyssönen et al., 2014, [26] Onstott et al., 2009, [27] Osburn et al., 2014, [28] Pedersen and Ekendahl, 1990, [29] Pedersen and Haveman, 1999, [30] Pitkänen and Partamies, 2007, [31] Pitkänen et al., 1996, [32] Pitkänen et al., 1998, [33] Purkamo et al., 2013, [34] Purkamo et al., 2015, [35] Rajala et al., 2015, [36] Sherwood Lollar et al., 2006, [37] Stotler et al., 2009, [38] Stotler et al., 2010, [39] Ward et al., 2004*.

The emerging interest in using stable bedrock formations for industrial purposes (e.g., nuclear waste disposal sites or carbon dioxide or natural gas storages) has increased the need for understanding the complete carbon cycling scheme in deep crystalline rock environments. CH_4_ can have effects on industrial utilization of the deep bedrock: It can promote growth of microorganisms in the subsurface by providing an ample source of energy and carbon. Increased microbial activity can lead to pH changes inducing corrosion and alteration of geochemistry resulting to mineral precipitation and scaling, or mobilization of hazardous compounds such as radiocarbon, thus damaging the infrastructure or imposing a risk to the environment. Therefore, research has been conducted for several years for example related to the long-term geological disposal of spent nuclear fuel to deep subsurface facilities in Canada, Sweden, USA, Finland, and Belgium (Stroes-Gascoyne and West, 1996; Pedersen, 1996; Fredrickson et al., 2004; Wang and Francis, 2005; Nyyssönen et al., 2012; Wouters et al., 2013). These studies have provided not only an understanding of the microbial risks, but also knowledge of the active microbial communities living in deep geological surroundings. However, many aspects of origin, source, and cycling of CH_4_ in deep continental bedrock environments still remain poorly understood. These include sources of carbon and hydrogen in different rock formations, importance of microbial involvement, timescales and quantities of CH_4_ production, and movements within and fluxes from bedrock.

**Figure 1:**
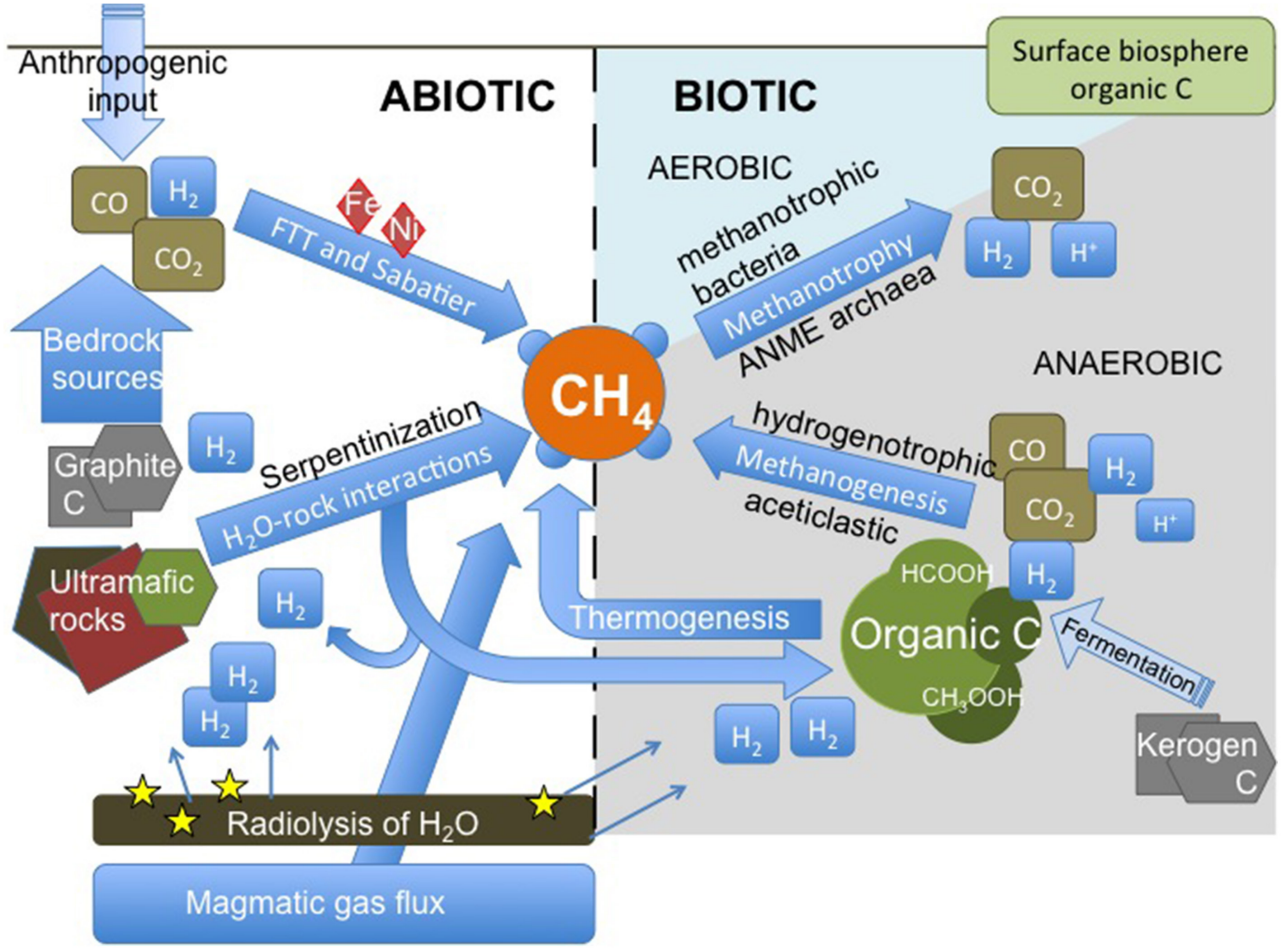
**Methane cycling scheme in the deep continental biosphere**. Methane can originate either from low temperature abiotic reactions in the upper crust, as a gas flux deeper from the crust or mantle or as a result of microbial activity. CO_2_ and organic matter are common carbon sources, while hydrogen can be derived from breakdown of water in radiolysis, from water-rock interactions or from microbial metabolism. Biological consumption of CH_4_ can be divided to aerobic and anaerobic methane oxidation, the former being more abundant in shallower depths and the latter in greater depths. Hydrogenotrophic methanogens use inorganic carbon for the production of CH_4_, as aceticlastic or methylotrophic methanogens use organic carbon molecules, such as formate or acetate. Bacterial fermentation of complex carbon-containing materials, such as kerogen, may produce hydrogen and small organic molecules for methanogens. FTT, Fischer-Tropsch type synthesis of hydrocarbons; ANME archaea, anaerobic methanotrophic archaea.

Potential formation pathways of CH_4_ in deep crystalline bedrock are presented in Figure 1. In principle, hydrocarbons can form via two major ways: from break-up of organic matter (“from big to small”) or due to organic synthesis of small C and H bearing molecules and further polymerization of these molecules into higher hydrocarbons (“from small to big”). The first way includes the formation of thermogenic CH_4_ and microbial aceticlastic methanogenesis. The latter includes abiotic organic synthesis, such as Fischer-Tropsch or Sabatier type reactions, and microbial hydrogenotrophic methanogenesis. Thus, both ways can be facilitated by microorganisms but may also occur inorganically. Not only is CH_4_ produced but is also consumed within bedrock by anaerobic and aerobic processes with and without microorganisms.

The occurrence and major sources for abiotic CH_4_ was discussed in a recent review by Etiope and Sherwood Lollar (2013) and microbial biodiversity in terrestrial subsurface environments by Fredrickson and Balkwill (2006). Yet it has been over a decade since the microbial CH_4_ cycling in deep subsurface has been reviewed (Kotelnikova, 2002). Since then the scientific community has been pushing the understanding of deep carbon cycling to new frontiers. Recent advances in molecular biological methods, such as high-throughput sequencing and “omics” methods have expanded the knowledge of microbial community composition and functions in the deep biosphere (Teske and Biddle, 2008; Brazelton et al., 2012; Nyyssönen et al., 2014). The development of new computational methods for processing, mining and assessing metagenomic data has provided tools for handling the vast amount of data generated (e.g., Schloss et al., 2009; Caporaso et al., 2010; Langille et al., 2013). Some novel methods have been used to assess the microbial ecology of the deep terrestrial subsurface. For example, a single-species ecosystem fully independent from surface life and photosynthesis, gaining energy from sulfate reduction was discovered at 2.8 km depth from South African gold mine in 2008 by Chivian et al. with metagenomics approach (Chivian et al., 2008). Nyyssönen et al. (2014) used high-throughput amplicon sequencing and metagenomics for characterization of the 2.5 km Outokumpu Deep Drill Hole microbial communities in Finland.

In this review we aim to revise methane cycling within continental bedrock, as well as to provide an overview of methodological and conceptual advances in this field with an emphasis on microbiology. Diversity and variation of geochemical and microbiological characteristics in CH_4_ containing deep bedrock environments are introduced through several case studies. Finally, we point out some key remaining unknowns and give suggestions for further research.

## Geological methane

Two main types of geological CH_4_ are depicted in Figure 1 which summarizes CH_4_ cycling in the continental deep biosphere. Of these, thermogenic CH_4_ is formed from the break-up of organic matter at elevated temperatures and pressures. This is thought to be the dominant CH_4_ type in sedimentary basins and is the economically most important source of natural gas (e.g., Galimov, 1988; Milkov, 2011). However, even though some rocks found in continental shields, most notably black schists, also originate from organic rich material, the occurrence and preservation of “fossil” organic compounds in highly metamorphosed crystalline rocks is questionable (but see Karus et al., 1987 and Taran et al., 2011 for examples of organic compounds such as bitumen found in metamorphic and even igneous rocks of Archaean to Proterozoic age). Instead, carbon is mostly found in the form of graphite and carbonate minerals. Thus, and taking into account the low temperatures presently prevailing in continental shield sites (Table 1), any thermogenic CH_4_ component would likely be relic. Where hydrocarbon-bearing sedimentary formations are nearby, thermogenic CH_4_ may find its way into metamorphic and igneous rocks by diffusion or advective flow (Etiope and Martinelli, 2002 and references therein).

Probably the more important source of geological CH_4_ in crystalline bedrock environments is abiotic. This refers to CH_4_ that has formed from inorganic compounds without the involvement of biological activity. Abiotic CH_4_ can be either magmatic or produced in water-rock reactions, the latter of which may take place even at low temperatures and pressures (Etiope and Sherwood Lollar, 2013). Within the habitable zone in the upper crust, high temperature abiotic CH_4_ may be provided by a gas flux from deeper, hotter regions (Figure 1) or leak from fluid inclusions. However, abiotic CH_4_ can also potentially form *in situ* in low temperature processes which include Sabatier and Fischer-Tropsch type (FTT) synthesis of CH_4_ (Jacquemin et al., 2010; McCollom et al., 2010; Etiope and Sherwood Lollar, 2013; Zhang et al., 2013). These reactions are most likely catalyzed by metals, such as Fe and Ni or certain mineral phases, such as clay minerals (Jacquemin et al., 2010; McCollom et al., 2010; McCollom, 2013; Etiope and Ionescu, 2014). More detailed description of the possible mechanisms of abiotic CH_4_ formation can be found in recent reviews by Etiope and Sherwood Lollar (2013) and McCollom (2013).

## Microbial contribution to CH_4_ budget in the bedrock

### Methanogenesis

Microbial methanogenesis is constrained to the domain Archaea. Methanogens can be divided to two groups depending on their CH_4_ production pathways: chemolithoautotrophic methanogens utilizing solely CO_2_ and H_2_ for their cellular building blocks and energy production and organotrophic methanogens utilizing an array of different carbon molecules containing methyl group such as acetate, methanol, methylamines, and methylsulfides as substrates (Figure 1) (e.g. Garcia et al., 2000; Thauer et al., 2008; Ferry, 2010, 2011).

Methanogens thrive in many environments considered extreme from an anthropocentric point of view. These include deep, dark, isolated, and nutrient-depleted subsurface environments. Typically methanogenic archaea can be found in anaerobic environments where all other electron acceptors but CO_2_ are limiting. Methanogenic archaea are an essential part of the microbial communities in deep continental crystalline biosphere as several studies from over the last 20 years have demonstrated (Table 1 and e.g., Sherwood Lollar et al., 1993a; Kotelnikova and Pedersen, 1997, review by Kotelnikova, 2002).

Today, seven methanogenic archaeal orders are known: *Methanopyrales, Methanococcales, Methanobacteriales, Methanocellales*, and *Methanomicrobiales* (all of which depend on H_2_ and CO_2_ and some of them can utilize formate as carbon source) and *Methanosarcinales* (with more versatile carbon metabolism) (Thauer et al., 2008; Costa and Leigh, 2014). In addition to these, the representatives of the recently proposed seventh order “*Methanoplasmatales*” are methylotrophic methanogens utilizing methanol, methylamines, and H_2_ (Paul et al., 2012; Borrel et al., 2013). Methanogenesis involves several enzymes and cofactors, resulting in a complex metabolic process.

In all methanogenic pathways, the final step in production of methane is performed by methyl-coenzyme M reductase. The gene *mcr*A, coding for the alpha subunit of this enzyme, has been used widely as a marker gene for methanogenesis in various environmental studies (e.g., Luton et al., 2002; Chin et al., 2004; Dhillon et al., 2005; Juottonen et al., 2006). Heterodisulfide reductase, which is required in the final step of methanogenesis, can be either membrane-bound (*Methanosarcinales*) or cytoplasmic (other methanogens). It has been observed that *Methanosarcinales* with membrane bound cytochromes have higher growth yields but methanogens with cytoplasmic heterosulfide reductase enzyme have lower threshold level for H_2_ partial pressure. Consequently, methanogenesis from carbon dioxide is dependent on hydrogen ion concentration, while electrons from the methanogenesis process are bound to hydrogen ions to drive ATP synthase and ultimately produce the energy for the methanogens (Thauer et al., 2008). Due to the lower threshold level for H_2_, hydrogenotrophic methanogens can outcompete *Methanosarcinales* -methanogens in environments with low hydrogen concentration. There are more hydrogenotrophic methanogens than methanogens utilizing methylotrophic or aceticlastic pathways (Liu and Whitman, 2008). Whether this is because of the hydrogenotrophic CO_2_-pathway is more favorable energetically, or hydrogenotrophic pathway being more ancient and thus has had more time to spread and diversify in the archaeal populations, or there has been more success in cultivating hydrogenotrophic methanogens, is still debatable.

### Methane oxidation

In addition to methane-producing archaea, the deep subsurface environments host microbes utilizing CH_4_ for their sole source of carbon and energy, called methanotrophic microorganisms (Figure 1). Electron acceptors can vary from oxygen to sulfate, nitrate and nitrite, iron and manganese (Hanson and Hanson, 1996; Orphan et al., 2002; Raghoebarsing et al., 2006; Beal et al., 2009; Knittel and Boetius, 2009; Ettwig et al., 2010; Haroon et al., 2013). As most of the deep crystalline bedrock habitats are mainly anaerobic, other electron acceptors than oxygen could be more relevant in these environments. Nevertheless, the detection of both aerobic and anaerobic methanotrophs from deep crystalline bedrock prove that both of these microbial groups have a niche in the depths (Table 1) (Kalyuzhnaya et al., 1999; Chi Fru, 2008; Hirayama et al., 2011; Nyyssönen et al., 2012; Bomberg et al., 2015; Purkamo et al., 2015; Rajala et al., 2015). In addition, aerobic methylotrophs appear to frequently occupy geological lignite and coal formations that are usually considered anaerobic, at depths of over 1 km (Mills et al., 2010; Stępniewska et al., 2013, 2014). Anaerobic methanotrophic archaea (ANME archaea) are frequently found from deep subseafloor sediments (e.g., Mills et al., 2003; Knittel et al., 2005; Lazar et al., 2011a,b) and even in deeply buried Juan de Fuca Ridge flank basalts (Lever et al., 2013). They are more rarely encountered in continental deep biosphere. However, ANME archaea have been recently detected in groundwaters in Olkiluoto of the Fennoscandian Shield, Finland (Nyyssönen et al., 2012; Bomberg et al., 2015).

#### Aerobic methane oxidation

Representatives of aerobic methanotrophic bacteria can be found from alpha- and gamma-proteobacterial families and class *Verrucomicrobiae*. Aerobic methanotrophs use two types of biosynthesis pathways for incorporation of methane, either serine pathway or ribulose monophosphate pathway (RuMP) (Chistoserdova et al., 2009; Nazaries et al., 2013).

The aerobic methanotrophic bacteria have distinct intracellular membrane features. These intracellular membranes are arranged as stacks of disc-shaped features or as paired membranes following the brim of the cell interior. Methane monooxygenase, the key enzyme of aerobic CH_4_ oxidation is located in these intracellular membrane structures. Methane monooxygenase mediates the first step of methanotrophy by oxidation of CH_4_ to methanol. Two types of methane monooxygenases exist: membrane-bound particulate MMO, which is the common type and soluble cytoplasmic MMO that is found irregularly in a few methanotrophic strains. Soluble methane monooxygenase can oxidize a wide range of different types of substrates from simple alkanes to cyclic compounds, thus evoking industrial interest (Bowman, 2006).

#### Anaerobic oxidation of methane (AOM)

All but one currently recognized anaerobic methanotrophs are archaea. The single exception is a bacterium *Methylomirabilis oxyfera*, which will be discussed in the next paragraph. Anaerobic methanotrophic archaea often referred to as ANME archaea usually live in a symbiotic relationship with sulfate reducers and are therefore abundant in sulfate-methane transition zones (SMTZ). SMTZs can be found in all anaerobic aquatic ecosystems where sulfate is available, such as marine sediments and deep terrestrial crystalline bedrock formations (e.g., Knittel and Boetius, 2009; Bomberg et al., 2015). ANME-1 and ANME-2 groups are usually associated with deltaproteobacterial *Desulfosarcina* and *Desulfococcus*. There is limited evidence that some ANME-2 archaea can form symbiotic relationships with alpha-proteobacterial *Sphingomonas* sp. or with betaproteobacterial *Burkholderia* sp. (Orphan et al., 2001, 2002; Knittel and Boetius, 2009). In addition, ANME-2d archaea have been demonstrated to oxidize CH_4_ and reduce nitrate in co-culture with an anaerobic ammonia oxidizer *Kuenenia* sp. (Haroon et al., 2013). Iron and manganese oxide minerals can be used as electron acceptors for anaerobic methane oxidation (Beal et al., 2009). ANME-3 archaea are typically associated with *Desulfobulbus* -type of sulfate reducers (Knittel and Boetius, 2009).

The only bacterial representative of an anaerobic methane oxidizer was found in anaerobic sediment from a Dutch canal and is candidatively named *Methanomirabilis oxyfera* (Ettwig et al., 2010). These peculiar bacteria grow anaerobically reducing nitrite to dinitrogen by nitric oxide dismutation simultaneously producing oxygen as an intermediate during this process within the cell. Oxygen is then used in the aerobic oxidation of CH_4_ to methanol with methane monooxygenase inside the cell.

The biochemical mechanism of anaerobic methane oxidation remains elusive. One hypothesis is that ANME archaea are reversing the methanogenic pathway and taking into use the key enzymes of this pathway. Released hydrogen from this process is removed by reducing electron acceptors such as sulfate by the syntrophic partner of ANMEs (Hallam et al., 2004; Knittel and Boetius, 2009). Results from metagenomic studies have fortified the reverse methanogenesis hypothesis (Krüger et al., 2003; Meyerdierks et al., 2005). Another suggested mechanism is acetogenic methanotrophy, in which acetate and hydrogen are produced from two molecules of CH_4_, or from CO_2_ and CH_4_ and subsequently consumed by sulfate reducing bacteria (SRB). Yet another proposed hypothesis involves CO_2_ reducing, methane-oxidizing archaea producing methyl sulfides following a pathway similar to methanogenesis. In this methylogenesis model, methyl groups are transferred to sulfide allowing the regeneration of coenzyme M. SRB capable of utilizing methyl sulfides are crucial partner in these type of AOM consortiums (review by Caldwell et al., 2008 and references within). While SRB have in general a significant role in AOM, a recent study by Milucka et al. (2012) showed that methanotrophic ANME-2 archaea can perform dissimilatory sulfate reduction without the syntrophic sulfate-reducing bacterial partner.

## Origin of substrates for methanogenesis in deep crystalline rock biosphere

Biological production and consumption of CH_4_ in deep crystalline bedrock is illustrated on the right in Figure 1. Abiotically produced carbon dioxide and hydrogen provide a useful source of carbon and energy for chemolithoautotrophic methanogens (Pedersen, 1997, 2000; Chapelle et al., 2002; Nealson et al., 2005; Schrenk et al., 2013). Within crystalline bedrock H_2_ needed for autotrophic CH_4_ synthesis can be produced during water-rock interactions such as serpentinization and other iron oxidation reactions (McCollom and Bach, 2009; Neubeck et al., 2011; Mayhew et al., 2013). It can also be produced through the dissociation of water molecules by energy released from radioactive decay, i.e., in radiolysis (Vovk, 1987; Lin et al., 2005a,b). The source of hydrogen may also be microbial (reviews by e.g., Nandi and Sengupta, 1998; Wang and Wan, 2009). Fermentative bacteria producing H_2_, such as clostridia have been detected in several deep continental rock formations (e.g., Moser et al., 2003; Rastogi et al., 2010; Itävaara et al., 2011b; Purkamo et al., 2013; Schrenk et al., 2013). Overall, H_2_ is common in these environments (Sherwood Lollar et al., 2014). Inorganic carbon can be found as carbonate minerals, graphite, and dissolved in groundwater (DIC). The speciation of DIC is controlled by pH. In alkaline conditions, typical of deep groundwaters within crystalline rocks, DIC is commonly found in the form of bicarbonate (HCO^−^_3_) and the concentrations may be quite low (Table 1).

In addition to inorganic carbon, methanogens can utilize organic compounds possibly produced in abiotic reactions, such as serpentinization of olivine-bearing ultramafic rocks (Lang et al., 2012). This principle can be turned inside out; in a recent review, Schrenk et al. (2013) suggested that heterotrophs might be the primary producers in serpentinizing environments. Thus, these heterotrophs utilizing organic materials (produced in abiotic reactions in deep Earth's crust or mantle) biologically produce inorganic end-products such as H_2_ and CO_2_. These can be used by organisms considered autotrophic, such as hydrogenotrophic methanogens.

Methanogenic archaea can also have the same function in the deep biosphere as in surface ecosystems, i.e., contributing to the degradation of organic matter in anaerobic conditions. Crystalline rock formations may contain refractory organic carbon materials such as kerogen or bitumoids for example in interlayers of black shales or black schist (Karus et al., 1987; Taran et al., 2011). During the formation of these rocks through diagenesis and maturation of sedimentary organic matter, carbon content, and aromaticity increase and volatile hydrocarbons usually migrate away (e.g., Strąpoć et al., 2011; Buseck and Beyssac, 2014). Further increase in temperature during metamorphosis will eventually turn the residual carbonaceous matter into graphite.

There are reports of microbes utilizing organic material trapped in sedimentary rocks such as shales and sandstones. Kerogen of black shales can be the sole carbon source for heterotrophs, such as *Clostridium* -type of bacteria, which in turn can support methanogenesis through the production of substrates for methanogens (Krumholz et al., 1997; Petsch et al., 2001, 2005). For example, clostridial fermenters can provide H_2_ and carbon to methanogens in a syntrophic consortium (Kimura et al., 2010; Rosewarne et al., 2013a,b). Thus, methanogens living in a syntrophic relationship with specialized bacteria can mediate anaerobic biodegradation of refractory complex compounds comparable to those found within crystalline bedrock (Strąpoć et al., 2011 and references within).

Fungal contribution to degradation of refractory material, such as organic polymers and polyaromatic hydrocarbons is well known (e.g., Haritash and Kaushik, 2009; Harms et al., 2011). In addition, fungal degradation of refractory organic matter of black shale has been demonstrated (Wengel et al., 2006). As the presence of fungi in deep Fennoscandian bedrock has been verified (Ekendahl et al., 2003; Sohlberg et al., 2015), fungi with the capacity for degradation of refractory and ancient organic and inorganic materials and efficient elemental cycling in addition to bioweathering ability might play a role in providing carbon sources for microbial methanogenesis in deep continental crystalline bedrock environments.

Organic matter from dead microbial biomass is an additional source of carbon in deep biosphere. Bacteriophages are present in deep crystalline bedrock groundwater. If these viruses are active and lytic, they can control the numbers of living microorganisms and therefore increase the dead cell mass in the deep subsurface (Kyle et al., 2008). Recently, Pedersen (2013) concluded that bacteriophages control the cell numbers in flow cells operating in *in situ* conditions in deep crystalline bedrock. The cell number in the flow cells never exceeded the cell densities observed in pristine groundwater (Pedersen, 2013).

Carbon sources for biological CH_4_ cycling can be anthropogenic and derived from the surface environments, such as in deep subsurface storage facilities for carbon dioxide or oil. Introduction of such carbon sources to microbial communities in the deep subsurface might induce a formidable activation of dormant microbes as Rajala et al. (2015) demonstrated. H_2_ can be released through a chemical reaction between freshly crushed rock and water for example during drilling and blasting of rock (Kita et al., 1982), or oxidation of metals such as iron casing which is often used to support drill hole walls.

To summarize, H_2_ seems to be abundant and available in continental crust (Sherwood Lollar et al., 2014). Despite the several possible sources of organic carbon in the crystalline rock formations discussed above, organic carbon is either absent, scarce or available only as a refractory material (Fredrickson et al., 1997; Fredrickson and Balkwill, 2006). Likewise, concentrations of dissolved inorganic carbon in deep bedrock formations are commonly low (Table 1). Thus, it might be carbon which actually limits the formation of CH_4_ and other hydrocarbons in continental bedrock.

## Geochemical methods to study origin, source, and cycling of CH_4_ - principles and limitations

### Stable isotope composition of CH_4_

The attempts to separate different CH_4_ sources are mainly based on the isotopic composition of CH_4_. Typically this includes the determination of stable isotope ratios of carbon (^13^C/^12^C) and hydrogen (^2^H/^1^H, also called D/H where D stands for deuterium) separately. The resulting isotopic compositions are commonly reported using δ-notation per mill (‰) relative to the isotopic composition of H and C in sea water (VSMOW, Vienna Standard Mean Ocean Water and VPDB, Vienna Pee Dee Belemnite):

$$\begin{array}{l} \delta^{2}\text{H}=\left(\frac{{{(}^{2}H{/}^{1}H)}_{sample}}{{{(}^{2}H{/}^{1}H)}_{VSMOW}}-1\right)\times 1000\\ \delta^{13}C=\left(\frac{{{(}^{13}C{/}^{12}C)}_{sample}}{{{(}^{13}C{/}^{12}C)}_{VPDB}}-1\right)\times 1000 \end{array}$$

Fundamentals of stable isotope methods in geochemistry can be found from books by Clark and Fritz (1997) and Hoefs (2004). In brief, the isotopic composition of CH_4_ (or any chemical substance) is controlled by equilibrium and kinetic isotope effects, which arise from equilibrium isotope exchange and differences in reaction rates, respectively. Equilibrium isotope effects are mainly dependent on temperature. Kinetic fractionation is related to incomplete and unidirectional reactions such as those associated with microbial metabolism, and will lead to the depletion of lighter isotopes (^12^C and ^1^H) in the reaction product. These principles also form the basis for traditional classification of CH_4_with δ^13^C vs. δ^2^H diagram (Schoell, 1980; Whiticar, 1999; Etiope and Sherwood Lollar, 2013; Etiope and Schoell, 2014).

The isotopic composition of CH_4_ is ultimately dependent on the starting material(s) and is further affected by microbial activity, openness of the system, temperature and time, to name a few. Along with increasing amount of data from various settings, it has become clear that CH_4_ from different origins may have similar isotopic composition. For example, unusual ^13^C enriched microbial CH_4_ has been found from saline substrate limited environments (Kelley et al., 2012; Tazaz et al., 2013). Carbon limited conditions were also thought to be responsible for ^13^C enriched metabolic products found from the Lost City Hydrothermal Field by Bradley et al. (2009). Valentine et al. (2004a) found that the fractionation associated with methanogenesis was correlated with temperature and metabolic rate. Likewise, abiotic CH_4_ may have a wide range of isotopic compositions. When compared to both microbial and thermogenic CH_4_, abiotic CH_4_ is typically enriched in ^13^C (Etiope and Sherwood Lollar, 2013). However, abiotic CH_4_ produced in laboratory experiments has been rather depleted in ^13^C, down to around −50 ‰ VPDB (Horita and Berndt, 1999; Taran et al., 2007).

Difference in the source of hydrogen (methyl group vs. water) forms the basis for separating aceticlastic from autotrophic microbial CH_4_ by means of hydrogen isotopic composition of CH_4_ (Sugimoto and Wada, 1995; Whiticar, 1999). Hydrogenase enzymes are known to rapidly equilibrate the isotopic compositions of H_2_ and H_2_O (Sugimoto and Wada, 1995; Valentine et al., 2004b). Furthermore, hydrogen isotope fractionation in the system CH_4_-H_2_O-H_2_ can be used as a thermometer in a system where isotopic equilibrium has been attained, or it may help to reveal the amount of kinetic fractionation caused by biological processes (Bradley and Summons, 2010; Suda et al., 2014).

After formation, the isotopic composition of CH_4_ may change as the result of isotope exchange and equilibration, kinetic fractionation by abiotic or microbial oxidation (either aerobic or anaerobic) or migration (e.g., Coleman et al., 1981; Whiticar, 1999; Etiope et al., 2011). Compared to carbon, information on the CH_4_ source carried by hydrogen may be more easily lost by isotope exchange, especially when geological time scales are considered (Ni et al., 2011; Reeves et al., 2012). A further complication is brought up by mixing of CH_4_ originating from different sources.

### Co-existing CO_2_ and C_2+_ hydrocarbons

CO_2_ is usually a minor constituent in deep groundwaters within crystalline bedrock and, because of high pH typical for these environments, is mainly found in its dissolved form as HCO^−^_3_ (e.g., Clark and Fritz, 1997; Frape et al., 2003). Nevertheless, considering CO_2_ is a potential carbon source for both microbial and abiotic CH_4_, it would be essential to know its isotopic composition (or the isotopic composition of DIC at least) in order to study the origin of CH_4_. ^13^C depleted DIC has also been used as an indication of microbial CH_4_ oxidation as kinetic fractionation caused by this process will favor depletion of ^13^C in the product CO_2_ (Kotelnikova, 2002; Onstott et al., 2006). When recorded in minerals, such as calcite, this isotopic shift has been used for tracing carbon cycling in the past (e.g., Schidlowski, 2001; Drake and Tullborg, 2009; Sahlstedt et al., 2010).

Information on the origin and cycling of CH_4_ may also be obtained by comparing the abundance of CH_4_ to longer chained (“higher” or C_2+_) hydrocarbons. Longer chained hydrocarbons most commonly found in deep groundwaters within crystalline rocks are ethane and propane (Fritz et al., 1987; Nurmi et al., 1988; Sherwood Lollar et al., 1993a,b; Haveman et al., 1999; Ward et al., 2004; Kietäväinen et al., 2013). As microbial processes produce almost solely CH_4_ and not higher hydrocarbons, the ratio between CH_4_ and C_2+_ is very high (>10^3^), while CH_4_ produced by thermogenic break-up of organic matter tends to contain significant amounts of C_2+_ compounds (e.g., Whiticar, 1999). Hydrocarbon formation due to abiotic organic synthesis at geologically relevant conditions is more poorly constrained (McCollom, 2013). Formation of longer chained alkanes and alkenes is a typical feature of FTT synthesis (e.g., Taran et al., 2007; McCollom et al., 2010; Zhang et al., 2013). However, no longer chained hydrocarbons were formed in the low temperature (298 K) FTT experiment conducted by Jacquemin et al. (2010) using CO_2_ and H_2_ as starting materials. The study by Horita and Berndt (1999) also suggest that the CH_4_ to C_2+_ ratio of abiotic gas might be closer to that of microbial than thermogenic gas. To summarize, variation in the CH_4_ to C_2+_ ratio of abiotic gas should be expected. Moreover, mixing of gases originating from different sources as well as microbial and inorganic reactions are capable of modifying the CH_4_ to C_2+_ ratio. For example, the ratio will be increased by both abiotic and microbial oxidation of CH_4_. Decrease in the ratio will take place if longer hydrocarbons are consumed in microbial CH_4_ production (Zengler et al., 1999).

A further approach is to compare the isotopic compositions of longer chained hydrocarbons and CH_4_. In typical thermogenic gas, the isotopic composition of carbon proceeds toward more ^13^C depleted compositions in the series propane-ethane-methane while isotopic depletion of ^13^C along with increasing chain length (reversed pattern) has been suggested to characterize abiotic hydrocarbons (e.g., Sherwood Lollar et al., 2002; Zhang et al., 2013). Both trends can be explained by reaction kinetics, as lighter ^12^C will both break and react faster. However, similarly to the concentration data, isotopic trends obtained from laboratory scale production of abiotic hydrocarbons vary (McCollom, 2013). Furthermore, it is not uncommon to see patterns in natural samples which are somewhere between these two. Zhang et al. (2013) proposed that different trends could be related to thermal history. According to their study, cracking of earlier formed longer chained hydrocarbons with increasing temperature would produce a typical thermogenic pattern while the reversed carbon isotope trend could be preserved in decreasing temperature. Kinnaman et al. (2007) found that large isotopic enrichment of both ^2^H and ^13^C in the substrate was associated with aerobic microbial CH_4_ oxidation. Thus the isotopic pattern could likely be changed by microbial processes. They also found that the fractionation clearly decreased with increasing chain length and/or when the substrate became limiting. This has important implications for deep continental subsurface environments which are characteristically substrate-limited. There, isotope fractionation by microbial processes is expected to diminish and may even be absent if the substrate is completely consumed.

### Other co-existing gases

In addition to carbon containing gases, other co-existing gases such as noble gases and N_2_ can be used to trace the origin of CH_4_. A major drawback related to using co-existing gases is the possible decoupling of these gases and CH_4_.

As they are inert, noble gases are very useful in tracing gas migration. For example they may be used to distinguish between mantle and crustal sources of gases (Kipfer et al., 2002; Prinzhofer, 2013; Sano and Fischer, 2013). Noble gases have also been used to determine residence times of deep groundwaters within crystalline shields in Canada (Bottomley et al., 1990; Greene et al., 2008; Holland et al., 2013), Fennoscandia (Kietäväinen et al., 2014; Trinchero et al., 2014), and South Africa (Lippmann et al., 2003). These studies have revealed ancient fluids within these formations extending from several millions of years to over a billion years old. Even though the information on residence times obtained from noble gases is indirect, and often comes with high uncertainties, it can be potentially utilized in estimating timing and rates of CH_4_ production as well as isolation of the subsurface ecosystems. Examples of this method are included in the studies by Lin et al. (2006b) and Schlegel et al. (2011).

Isotopic composition of N_2_ in groundwaters of the Fennoscandian and Canadian shields was used by Sherwood Lollar et al. (1993a) to show that hydrocarbons were not related to shallow atmosphere derived fluids but originated from the crystalline basement. Attempts to separate between inorganic and organic sources of hydrocarbons may also benefit from determination of N_2_ isotopes, as ^15^N depleted values are suggested to be representative of organic origin (Sano et al., 1993; Zhu et al., 2000; Etiope et al., 2011). As N_2_ is the main constituent of air, atmospheric contamination during sampling or analysis is a real risk to be aware of.

### Radiocarbon

Attempts have also been made to estimate the age of CH_4_ by using radiocarbon (^14^C) dating. One such study was performed by Slater et al. (2006) among the deep continental bedrock sites in Witwatersrand Basin, South Africa. By comparing the ^14^C isotopic composition of DIC and CH_4_ they concluded that the majority of the CH_4_ was produced in the distant past. Potential problems of this method include the contamination of typically CO_2_-undersaturated groundwater samples by atmospheric CO_2_, and recent formation of CH_4_ from ancient (or “^14^C dead”) carbon source. In the former case, the apparent age of DIC may be underestimated, while in the latter case CH_4_ from on-going processes could be interpreted as ancient. Nevertheless, ^14^C determination could help with tracing the carbon source. For example, Stotler et al. (2010) found that the carbon source for CH_4_ in the Lupin mine in Canada was older than could be dated with ^14^C, i.e., more than 50 ka.

### Clumped isotopes

Recently, clumped isotope methods have also been developed which are capable of determining the isotopologues of CH_4_ molecules (Stolper et al., 2014; Wang et al., 2015). Potential applications of the method include determination of CH_4_ formation temperature (CH_4_ thermometry), and detection of kinetic isotope fractionation, both of which might be used in separating biotic from abiotic CH_4_ (Stolper et al., 2014; Wang et al., 2015). More applications are expected when this method comes more widely attainable.

## Microbes involved in methane cycling in the fennoscandian shield and other deep precambrian continental subsurface environments

### Outokumpu deep drill hole

Formation waters at the 2.5 km deep scientific drill hole in Outokumpu, eastern Finland are characterized by high salinity and abundant dissolved gas phase of which CH_4_ covers up to 80 vol-% (Table 1). The bedrock in Outokumpu is composed of several geochemically different rock types. These include serpentinites which have gained a lot of attention recently in the studies of abiotic CH_4_, along with black schists which contain abundant C. To date, Outokumpu Deep Drill Hole is the deepest site within the Fennoscandian Shield where CH_4_ cycling microbes have been studied.

Marker genes for methanogenesis, *mcr*A were detected throughout the drill hole water column to 1500 m depth with a quantitative PCR assay (Itävaara et al., 2011a). Thus the existence of methanogens in Outokumpu could be verified, even though the copy numbers in a ml of drill hole water were essentially low (around 1 × 10^2^ copies mL^−1^). The overall ratio of methanogens vs. total number of bacteria was less than 1%. Cloning of the methanogenesis marker gene from the drill hole water suggested that at depths of 900 m or shallower, aceticlastic *Methanosarcina* were present in the methanogenic communities. Methylotrophic *Methanolobus* -type of OTUs were detected by cloning and high-throughput sequencing methods from 1.1, 1.3, and 1.5 km depths (Nyyssönen et al., 2014; Purkamo et al., 2015). Lithology of the latter depths is dominated by serpentinites and black schists and statistically corresponds with the detection of *mcr*A genes of *Methanolobus* (Västi, 2011; Purkamo et al., 2015). Majority of the *mcr*A clones and pyrosequenced archaeal 16S rRNA OTUs from the deepest part of the drill hole (1.9 km depth and below) were related to *Methanobacterium* -associated methanogens (Nyyssönen et al., 2014; Purkamo et al., 2015). Further proof for autotrophic methanogenesis in the deepest part of the drill hole was received from the metagenome of the sample from 2.3 km depth, in which genes involved in autotrophic methanogenesis were detected (Nyyssönen et al., 2014).

The bedrock fracture zones represent different microbial community as the drill hole at the same depth. When observing the intrinsic archaeal communities in bedrock fracture zones at different depths of Outokumpu bedrock, Purkamo et al. (2013) confirmed that hydrogenotrophic *Methanobacteriaceae* were dominating the archaeal community in the fracture zones at 500 and 2260 m levels. In addition, a small part of the archaeal community at these depths contained aceticlastic *Methanosarcina*. The results were based both on DNA and RNA, thus it can be presumed that these methanogens were active in these fractures. Despite of confirmed methanogenic activity in Outokumpu, CH_4_ is ^13^C rich (Table 1). Thus, it cannot be classified as microbial within traditional limits for isotopic composition of biological CH_4_.

In addition to methanogens, the existence and activity of methanotrophic microbes in Outokumpu groundwater is established. By cloning of the marker gene for particulate methane monooxygenase enzyme (*pmo*A), Purkamo et al. (2015) demonstrated that aerobic methanogens are part of the microbial communities at 600, 900, and 1500 m depths of the drill hole water column. All clones in this study were affiliated with a γ-proteobacterial *Methylomonas methanica*. Rajala et al. (2015) verified that methanotrophs at 500 m fracture zone could be rapidly activated with CH_4_, methanol and sulfate. No ANME-associated *mcr*A was found in these studies, suggesting either that an another type of anaerobic methane oxidation pathway than reverse methanogenesis could be more likely in Outokumpu, or aerobic oxidation of CH_4_ by bacteria is more likely in Outokumpu, or the *mcr*A primers used in these studies do not detect ANME-type of *mcr*A.

### Olkiluoto

Olkiluoto in southwestern Finland is the future repository site for nuclear waste. The bedrock in Olkiluoto is comprised of migmatitic mica gneisses. Similarly to Outokumpu, the deep groundwater in Olkiluoto is anaerobic and saline (Nyyssönen et al., 2012). Concentration of CH_4_ is among the highest observed today from any Precambrian crystalline bedrock site (Table 1). The microbial communities in different parts and depths of Olkiluoto site have been under observation for several years. Traditional most probable number (MPN) cultivation methods have described methylotrophs in shallow depths and methanogens to the depth of at least 450 m. Methanogens were present in small numbers, 1 × 10^0^ from 1 × 10^1^ cells mL^−1^ (Pedersen et al., 2008). Using molecular biological methods, Nyyssönen et al. (2012) reported that the majority of *mcr*A clones acquired from shallow (< 400 m) depths of different drill holes in Olkiluoto fell within a metabolically diverse group of methanogens, namely *Methanosarcinales*. In addition, *Methanoregula boonei* of the *Methanomicrobiales mcr*A sequences were detected, while hydrogenotrophic *Methanobacteriales* -type of *mcr*A were found below 500 m depth. The *mcr*A copy numbers detected with qPCR varied from less than 200 copies to below detection limit mL^−1^ of groundwater. Apparently the archaeal communities are diverse in Olkiluoto but methanogenic archaea represent only a minority ranging from 10 to 0.4 % of the archaeal community (Bomberg et al., 2014).

Nyyssönen et al. (2012) detected *mcr*A of putative anaerobic methane oxidizers, i.e., ANME archaea in a single sample from 350 m depth. In addition, this sample had the highest number of *mcr*A gene copies, 660 copies mL^−1^. This depth is considered to be within the sulfate-methane transition zone in the Olkiluoto bedrock. A flow cell cultivation study provided further evidence of existence of ANME archaea in Olkiluoto (Pedersen, 2013). In addition, it was shown that the active archaeal communities at depths of from 300 to 800 m in Olkiluoto consisted of, among others, ANME-2D archaea (Bomberg et al., 2015). These findings of ANME archaea are further proof for the hypothesis of Pedersen et al. (2008) that the anaerobic oxidation of methane is an active microbial process in the Olkiluoto bedrock.

### Äspö, Laxemar, and Forsmark

Another important Fennoscandian shield sites where deep biosphere studies have been conducted are in Sweden. Probably the most famous is the Äspö Hard Rock Laboratory (HRL) that has been running since 1995, in addition to Laxemar-Simpevarp and Forsmark which have been suggested for the final repository sites for nuclear waste. Äspö HRL extends to 450 m depth in porphyritic granite/granodiorite bedrock, whereas Forsmark lithology comprises of granites and Laxemar-Simpevarp granites and quartz monzodiorite (Table 1). Both salinity and CH_4_ concentrations are much lower in all Swedish sites compared to Olkiluoto and Outokumpu (Table 1). To our knowledge, no information on isotopic composition of CH_4_ detected at the Swedish sites exists. Pedersen (1997) demonstrated that methylotrophic and aceticlastic methanogens dominated at shallower depths above 200 m, where more organic carbon was available. In the deeper depths where organic carbon content was lower, autotrophic methanogens were more frequent. Kotelnikova et al. (1998) isolated an autotrophic methanogen from Äspö groundwater, *Methanobacterium subterraneum*. Hallbeck and Pedersen (2008a, 2012) found low numbers of methanogens based on the most probable number method from samples of all these three sites.

In addition to methanogens, methanotrophs have also been detected from deep bedrock of Äspö and Forsmark. Clone libraries of methanotrophy marker gene *pmo*A were dominated by *Methylomonas* and *Methylocystis* (Chi Fru, 2008). *Methylomonas* and *Methylobacter* dominated enrichment cultures from Äspö groundwater from below 400 m depth (Kalyuzhnaya et al., 1999). To conclude, methanotrophs are detected approximately at the same depths in all Fennoscandian Shield sites, mainly above 1 km.

### Witwatersrand and other deep precambrian continental subsurface study sites

Another widely studied deep biosphere is located in the Witwatersrand Basin in South Africa. The geological formation is composed of quartzite and shale with overlying basaltic and andesitic rocks, on top of the schist basement (Ward et al., 2004). Several sites in the Witwatersrand Basin have been studied and methanogenic as well as ANME archaea have been detected with cloning of 16s rRNA gene (Takai et al., 2001; Ward et al., 2004; Moser et al., 2005; Lin et al., 2005a, 2006a,b; Gihring et al., 2006). CH_4_ is a common constituent of the gas phase in all of these sites (Table 1). A wide range of isotopic compositions have been reported, including ^13^C poor CH_4_. In the Beatrix mine methanogens have been detected from 718 to 1390 m depths (Ward et al., 2004). At the depth of 866 mbls (BE16) *Methanobacterium*—type of clones were most abundant, while other detected methanogens affiliated with aceticlastic *Methanosarcina* and *Methanosaeta* (Lin et al., 2006a). The archaeal communities in the Evander mine at the depth of 1950 m (EV818) were composed of *Methanosarcina, Methanosaeta* in addition to *Methanolobus* (Ward et al., 2004; Gihring et al., 2006). *Methanobacterium*-type hydrogenotrophic methanogens were found typically in deeper samples in the Witwatersrand Basin, such as from the Mponeng and Driefontein mines from over 2.7 km depth (Moser et al., 2005; Gihring et al., 2006). In the Driefontein mine site D8A, at more than 3 km depth the archaeal community comprised about 10% of the total microbial community and was dominated by *Methanobacterium* (Moser et al., 2005). *Methanobacterium* -affiliating 16s rRNA sequences were detected also from the Mponeng mine at 2825 m depth (Lin et al., 2005a). Aceticlastic methanogens were typically found in depths shallower than 2 km at the Witwatersrand Basin whereas hydrogenotrophic methanogens such as *Methanobacterium* dominated at deeper depths. Gihring et al. (2006) suggested that high temperature and reducing conditions were the determining geochemical factors for the occurrence of *Methanobacteriales* in the fractures of the Witwatersrand Basin.

Anaerobic oxidation of methane in the deep subsurface in the Witwatersrand Basin is plausible, while archaeal 16s rRNA clones similar to methanotrophic ANME-1 archaea in Driefontein dolomitic aquifer and the Evander mine sites have been detected (Gihring et al., 2006).

Although life in the deep biosphere has been characterized in several sites as discussed above, there are some studies from deep continental bedrock sites where methanogens and methanotrophs could not be detected. These include the subsurface groundwaters from Palmottu, Kivetty, Romuvaara, and Hästholmen areas in the Fennoscandian Shield (Haveman et al., 1999; Haveman and Pedersen, 2002). Methanogenic archaea have not been detected from Kloof mine in the Witwatersrand Basin (Takai et al., 2001; Ward et al., 2004; Kieft et al., 2005). Neither did Onstott et al. (2009) detect any archaea from the Canadian Shield site in the Lupin mine. These results might indicate either real absence of methane cycling microbes at the sites or could be due to extremely low numbers of cells and/or inadequate sensitivity of analytical methods or because acquiring representative samples at field conditions is often challenging.

## Conclusions and future prospects

The possibility of abiotic CH_4_ synthesis at low temperatures, together with findings of methanogenic microbes indicates that the formation of CH_4_ is an on-going process in deep Precambrian continental bedrock. In addition, the detection of aerobic, and recently also anaerobic methanotrophs deep within crystalline bedrock provide ecological evidence of microbial contribution to CH_4_ consumption in these environments. So far, the identified carbon sources of microbes range from inorganic CO_2_ to CH_4_ and other small organic carbon molecules, but there are intriguing hypotheses on microbial utilization of refractory organic carbon of minerals. Thus, the participation of heterotrophic microbes in carbon cycling in deep crystalline bedrock should not be dismissed.

As deep continental crystalline bedrock environments are commonly carbon-deprived, the traditional isotopic separation between biologically produced and abiotically produced CH_4_ can be difficult. When carbon sources are limited, the small amount of carbon available will be utilized without the preference for lighter isotope that is considered to happen in “normal” (surface) circumstances with abundant organic carbon. In addition, microbial activities, such as syntrophy or competition, especially in substrate-limited environments, can result to similar isotopic composition of CH_4_ produced in abiogenic reactions. Thus, further studies are needed especially considering the effect of syntrophy or competitiveness of microbial species, substrate availability and reaction rates on the isotopic composition of CH_4_. Furthermore, more studies on different forms of inorganic carbon (including minerals) available for both abiotic and biological organic synthesis will be advantageous.

Metabolically diverse methanoges were found at shallower depths while hydrogenotrophic methanogenesis appeared to be more common at greater depths throughout the sites. However, transition between the zones is not sharp and the depth varies among the sites. Considering the evolution of methanogenesis, the hydrogenotrophic pathway may be as old as life itself on Earth, while the capability to use acetate is considered to have evolved more recently during the Cambrian period (Costa and Leigh, 2014 and references within). As the oldest bedrock fluids are dated to be Precambrian (over 1 billion years old; Holland et al., 2013) it will be interesting to see if there are any methanogenic communities in these isolated fracture fluids, and will the hydrogenotrophic pathway dominate in these communities, as the hypothesis of the methanogenesis evolution implies. Similarly, further studies on depth dependence and the extent of isolation of different methanogens can shed light on the evolution of deep bedrock biosphere as well as CH_4_ cycling.

Growing industrial interest in utilizing deep rock formations as a natural resource such as mining of valuable metals or extracting shale gas, as storage for CO_2_, hydrocarbons or other fuels, as a part of infrastructure such as traffic tunnels, and production of geothermal heat and/or energy has increased the need for understanding the origin, source and cycling of CH_4_ in these environments. The natural state of the bedrock will be disturbed during these activities and release, production, and consumption of CH_4_ can affect industrial operations. CH_4_ may enhance biological activity by providing energy and carbon for microbial communities. In turn, this may increase the concentrations of reactive compounds such as hydrogen and sulfide, especially in the presence of SO_4_, and change pH of the system thus increasing the corrosion risk. As a gaseous component, CH_4_ can also be important in mobilization of radiocarbon.

In order to understand CH_4_ cycling at depths, and the role of microorganisms within it, it is important to study the environmental conditions such as reduction-oxidation potential, isolation, and availability of substrates of the particular site together with microbiology. Geochemical methods may help to identify biotic from abiotic sources of CH_4_. Yet, no common characteristics in terms of pH, T, depth, lithology, abundance, and isotopic composition of CH_4_ could have been determined which would serve as diagnostic tools for estimating importance of microbial contribution in CH_4_ cycle in these environments. However, potential of new findings exists in all continental regions as to date very few sites have been studied at great detail or even superficially. For example, such studies would be beneficial in some Canadian Shield sites where extensive geochemical data on CH_4_ is available and world's oldest isolated bedrock fluids have been identified. In Sweden, on the other hand, more detailed geochemical characterization, including isotopic analyses could be helpful. In addition, development of new geochemical and microbiological methods, such as clumped isotopes and high-throughput sequencing can open new opportunities also in this field.

### Conflict of interest statement

The authors declare that the research was conducted in the absence of any commercial or financial relationships that could be construed as a potential conflict of interest.
